# Salinomycin enhances doxorubicin sensitivity through reversing the epithelial-mesenchymal transition of cholangiocarcinoma cells by regulating ARK5

**DOI:** 10.1590/1414-431X20176147

**Published:** 2017-08-17

**Authors:** Z. Yu, H. Cheng, H. Zhu, M. Cao, C. Lu, S. Bao, Y. Pan, Y. Li

**Affiliations:** 1 Department of General Surgery, Qingdao Clinic Medical College, Nanjing Medical University, Qingdao, China; 2 Department of General Surgery, The Second People's Hospital of Lianyungang, Lianyungang, China; 3 Department of General Surgery, The Afflicted Drum Tower Hospital, Nanjing University Medical School, Nanjing, China; 4 Department of Gastroenterology, The Afflicted Drum Tower Hospital, Nanjing University Medical School, Nanjing, China

**Keywords:** Chemotherapy, Cholangiocarcinoma, Drug resistance, Doxorubicin, Salinomycin

## Abstract

Chemotherapy response rates in patients with cholangiocarcinoma remain low, primarily due to the development of drug resistance. Epithelial-mesenchymal transition (EMT) of cancer cells is widely accepted to be important for metastasis and progression, but it has also been linked to the development of chemoresistance. Salinomycin (an antibiotic) has shown some potential as a chemotherapeutic agent as it selectively kills cancer stem cells, and has been hypothesized to block the EMT process. In this study, we investigated whether salinomycin could reverse the chemoresistance of cholangiocarcinoma cells to the chemotherapy drug doxorubicin. We found that combined salinomycin with doxorubicin treatment resulted in a significant decrease in cell viability compared with doxorubicin or salinomycin treatment alone in two cholangiocarcinoma cell lines (RBE and Huh-28). The dosages of both drugs that were required to produce a cytotoxic effect decreased, indicating that these two drugs have a synergistic effect. In terms of mechanism, salinomycin reversed doxorubicin-induced EMT of cholangiocarcinoma cells, as shown morphologically and through the detection of EMT markers. Moreover, we showed that salinomycin treatment downregulated the AMP-activated protein kinase family member 5 (ARK5) expression, which regulates the EMT process of cholangiocarcinoma. Our results indicated that salinomycin reversed the EMT process in cholangiocarcinoma cells by inhibiting ARK5 expression and enhanced the chemosensitivity of cholangiocarcinoma cells to doxorubicin. Therefore, a combined treatment of salinomycin with doxorubicin could be used to enhance doxorubicin sensitivity in patients with cholangiocarcinoma.

## Introduction

Cholangiocarcinoma is a highly malignant tumor of the bile duct that arises from epithelial cells (1,2). It is the second most common primary hepatic carcinoma and accounts for 10–20% of primary liver cancers (1,2). Due to its high malignant potential and rapid development, as well as the absence of associated chronic liver disease, early diagnosis of cholangiocarcinoma remains difficult (3). Therefore, many patients are at the advanced or terminal stage when diagnosed. For those patients who are able to undergo radical resection, the risk of recurrence remains high, with a 5-year survival rate of less than 30% (4–7). While some recent breakthroughs in liver transplantation have occurred, few cholangiocarcinoma patients are suitable for, or have access to this expensive treatment option (8–10). In addition, liver transplantation is not the best treatment method for cholangiocarcinoma, as it does not prevent recurrence.

In this respect, chemotherapy may provide more sustained benefits in cholangiocarcinoma patients. However, the response rate to chemotherapy response remains low, primarily due to the development of drug resistance (chemoresistance) (11). Therefore, novel strategies to overcome chemoresistance are urgently required.

Many studies have shown that epithelial-mesenchymal transition (EMT) is involved in the development of chemoresistance (12–16). For example, the chemotherapy drug doxorubicin was shown to induce EMT in different types of cancer cells, including breast cancer, hepatocellular carcinoma (HCC), and pancreatic cancer (17–19), although the mechanism of doxorubicin-induced EMT is unclear. The EMT process has also been shown to be involved in maintaining cancer stem cell (CSC) properties, such as the ability for self-renewal and differentiation (20). Therefore, the role of CSCs as tumor-initiating cells and in recurrence following chemotherapy has attracted attention.

Salinomycin is an ionophore antibiotic that can selectively kill CSCs, and thus may be useful in cancer chemotherapy (21–23). Salinomycin may also target the EMT process in cancer cells (23). Indeed, this antibiotic was previously shown to suppress ZEB1, an important activator of EMT, and to reverse the EMT process in mantle cell lymphoma (24). It is also important to note that poor prognosis of cholangiocarcinoma is related to high expression of ZEB1, likely due to its ability to activate EMT (25). Furthermore, salinomycin treatment was previously shown to suppress the proliferation, invasion, and metastasis of mesenchymal-type endometrial CSCs (26). Based on these data, we hypothesized that salinomycin may be useful in cholangiocarcinoma therapy.

In this study, we aimed to investigate the efficacy of salinomycin in the treatment of cholangiocarcinoma, and to determine if it could reverse chemoresistance to doxorubicin.

## Material and Methods

### Cell culture

Two cholangiocarcinoma cell lines, RBE and Huh-28, were purchased from the Chinese Academy of Science Cell Bank (China). RBE cells were maintained in RPMI-1640 medium (Gibco, USA) with 10% fetal bovine serum (FBS; Gibco), while Huh-28 cells were cultured in Dulbecco's modified Eagle's medium (DMEM; Gibco) with 10% FBS (Gibco).

### Chemical reagents

Doxorubicin and salinomycin were purchased from Sigma-Aldrich (USA). E-cadherin, Vimentin, ARK5, and GAPDH primary antibodies for western blotting were purchased from Abcam (USA). ARK5 siRNA was obtained from Santa Cruz (USA). The anti-CD133 (FITC) antibody used for flow cytometric analysis was purchased from eBioscience (USA).

### Cell viability assays

Cells were seeded onto 96-well plates at a density of 3000 cells/well with 100 μL cell culture medium. After 24 h, the medium was replaced with medium containing 10% FBS and different concentrations of doxorubicin (0, 0.0625, 0.125, 0.25, 0.5, or 1 μg/mL) or salinomycin (0, 2.5, 5, 10, 20, or 40 μM), and cultivated in a 37°C, 5% CO_2_ incubator for 48 h. Cell viability was subsequently measured at 3 h using the Cell Counting Kit-8 (Dojindo Laboratories, Japan) according to the manufacturer's instructions. Absorbance at 450 nm was measured by an MRX II microplate reader (Dynex, USA).

### Western blot analysis

A total of 2×10^5^ cells were seeded onto 6-well plates. Cells were washed with ice-cold PBS and then lysed using RIPA buffer (Sigma, USA). The protein concentration was quantified using the BCA Protein assay kit (Thermo, USA). Proteins (40 μg/lane) were separated by SDS-PAGE and transferred to polyvinylidene difluoride membranes (PVDF; Millipore, USA) for western blotting. Antibodies against ARK5, E-cadherin, vimentin, and GAPD were used for detection of proteins at 1:1000 dilution.

### RNA interference

RBE and Huh-28 cells were transfected with ARK5 siRNA using Lipofectamine 2000. Opti-MEM transfection medium was replaced with complete culture medium 6 h after transfection, and the RBE or Huh-28 were incubated for the indicated times. All experiments were performed 72 h after transfection. Transfected cells were plated onto 96-well plates at a density of 3000 cells/well, allowed to adhere overnight, and then treated with doxorubicin (0, 0.0625, 0.125, 0.25, 0.5, or 1 μg/mL) or salinomycin (0, 2.5, 5, 10, 20, or 40 μM) prior to the subsequent experiments.

### Flow cytometric analysis of CD133 expression

RBE cells were trypsinized and analyzed by flow cytometry (Cytomics FC500, Bechman Coulter, USA) using the anti-CD133 antibody (FITC).

### Statistical analysis

Prism 5.0 software was used for statistical analysis. The experimental data were assessed by a two-tailed Student *t*-test and are reported as mean±SD. Statistical significance was accepted if P<0.05.

## Results

### Salinomycin increased doxorubicin chemosensitivity in cholangiocarcinoma cells

First, we investigated whether salinomycin could increase the sensitivity of cholangiocarcinoma cells to doxorubicin chemotherapy using a cell viability (CCK-8) assay. When using doxorubicin alone, the IC_50_ for RBE and Huh-28 cells were 3.703 and 1.841 μg/mL, respectively (Table 1). Salinomycin was not effective at inhibiting the viability of cholangiocarcinoma cells unless high doses were used; the IC_50_ was 132 μM for RBE cells and 80.31 μM for Huh-28 cells. However, combined salinomycin with doxorubicin treatment for 48 h resulted in a significant decrease in cell viability compared with doxorubicin or salinomycin treatment alone in RBE and Huh-28 cells (Figure 1). In addition, the combination index values for these two cholangiocarcinoma cell lines after 48 h were 0.261 and 0.43, respectively, indicating that doxorubicin and salinomycin displayed synergism when used together (Table 1). Therefore, salinomycin treatment increased the sensitivity of cholangiocarcinoma cells to doxorubicin.

**Table 1.** Results of the cell viability assay (IC_50_ values) following treatment with doxorubicin (DOX) and/or salinomycin (SAL) in RBE and Huh-28 cell lines.

Cell line	IC_50_ of SAL (μg/mL)		IC_50_ of DOX (μg/mL)		Combination index
	SAL	SAL+DOX	DOX	SAL+DOX	
RBE	132	4.772*	3.73	0.838#	0.261
Huh-28	80.31	1.968*	1.841	0.3452#	0.430

Data are reported as the mean.

* P<0.05 *vs* SAL;

# P<0.05 *vs* DOX. Statistical analysis was carried out with the two-tailed Student *t*-test.

**Figure 1.**
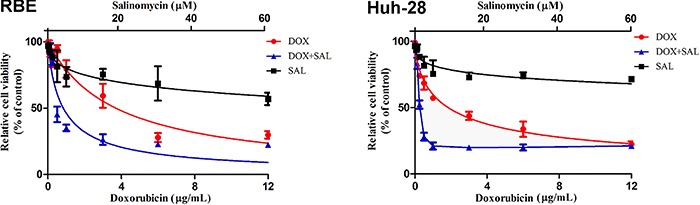
CCK-8 assay detection of the viability of RBE and Huh-28 cells following doxorubicin (DOX) and/or salinomycin (SAL) treatment. Salinomycin enhanced the effects of doxorubicin treatment on the cell viability of cholangiocarcinoma cells.

### Salinomycin reversed doxorubicin-induced EMT of cholangiocarcinoma cells

**Figure 2.**
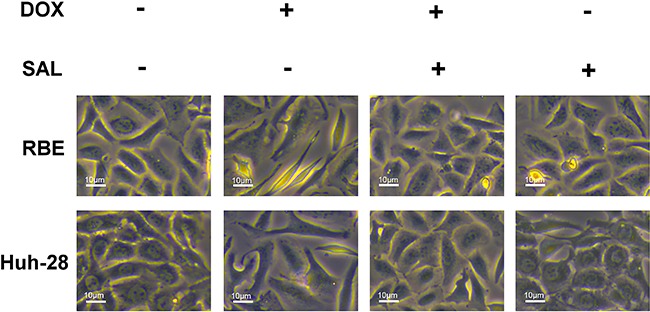
Morphological changes that occur when RBE and Huh-28 cells are cultured with doxorubicin (DOX) in the presence or absence of salinomycin (SAL) observed under bright field microscopy. Salinomycin reversed the effects of doxorubicin treatment on the morphology of cholangiocarcinoma cells.

To investigate the influence of salinomycin on the EMT process induced by doxorubicin treatment, we examined morphological changes and the expression of epithelial and mesenchymal markers in cholangiocarcinoma cells before and after doxorubicin treatment. Initially, both the RBE and Huh-28 cells were closely connected, polarized epithelial cells. However, after treatment with doxorubicin, both RBE and Huh-28 cells transformed into a diffuse fibroblast-like morphology. However, when treated with salinomycin alone, both RBE and Huh-28 cells maintained their original morphology. Furthermore, salinomycin treatment converted the diffuse fibroblast-like morphology observed with doxorubicin back to the closely connected, polarized morphology (Figure 2).

**Figure 3.**
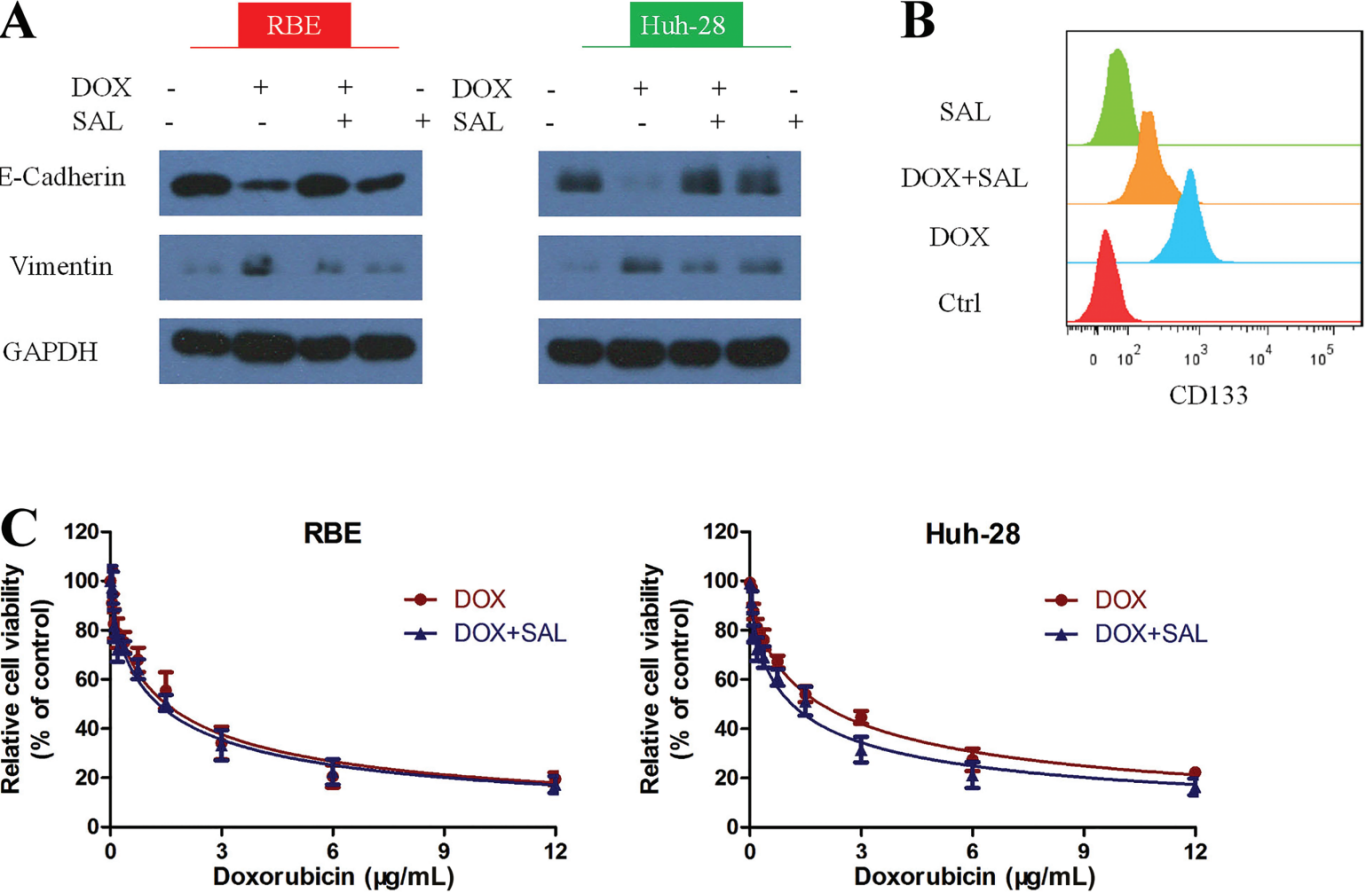
Salinomycin (SAL) reversed doxorubicin-induced epithelial-mesenchymal transition in cholangiocarcinoma cells. *A*, Western blot detection of E-cadherin and vimentin expression in control, doxorubicin- (DOX), doxorubicin plus SAL-, or SAL alone-treated cholangiocarcinoma cells. GAPDH was used as an internal control. *B*, Expression of CD133 detected by flow cytometry in RBE cells treated with DOX in the presence or absence of SAL. *C*, CCK-8 assay of the viability of RBE and Huh-28 cells following DOX and/or SAL treatment after twist siRNA interference.

We monitored the expression of EMT markers in RBE and Huh-28 cells via western blotting. Expression of the epithelial marker E-cadherin was lower when cells were treated with doxorubicin. However, when salinomycin was combined with doxorubicin treatment, E-cadherin expression increased. Similarly, doxorubicin treatment upregulated the expression of the mesenchymal marker vimentin in RBE and Huh-28 cells compared to the untreated control, whereas salinomycin reversed the doxorubicin-induced expression changes of vimentin (Figure 3A). Finally, we showed that after doxorubicin treatment, the expression of CD133 (a marker of CSCs) on RBE cells was increased, and when doxorubicin was combined with salinomycin, CD133 expression on RBE cells decreased (Figure 3B). Therefore, salinomycin reversed the doxorubicin-induced EMT of cholangiocarcinoma cells.

To further confirm that salinomycin could increase doxorubicin sensitivity toward cholangiocarcinoma cell lines through reversing EMT progress, we used twist siRNA to interfere in RBE and Huh-28 cells first, then treated both cells with doxorubicin or doxorubicin + salinomycin combination. We found that there was no significant difference between the two treatment methods (Figure 3C).

### Salinomycin reversed doxorubicin-induced EMT through regulating ARK5

**Figure 4.**
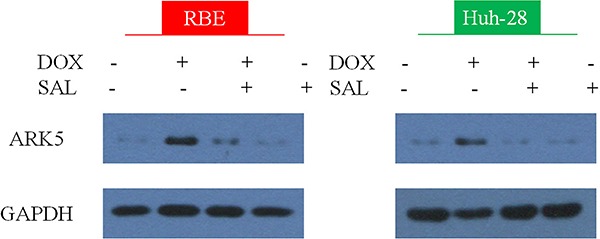
Salinomycin (SAL) reduced the doxorubicin-induced expression of AMP-activated protein kinase family member 5 (ARK5) in cholangiocarcinoma RBE and Huh-28 cells after treatment with doxorubicin (DOX), doxorubicin plus SAL, or SAL alone.

Overexpression of the AMP-activated protein kinase family member 5 (ARK5), a novel human AMP-activated protein kinase family member (27), was previously shown to decrease the sensitivity of HCC cells to doxorubicin. ARK5 promotes doxorubicin resistance in hepatocellular carcinoma via epithelial–mesenchymal transition (28). Therefore, we examined the expression of ARK5 in RBE and Huh-28 cells treated with doxorubicin, doxorubicin plus salinomycin, or salinomycin alone for 48 h. Doxorubicin treatment significantly upregulated expression of ARK5 in both cell lines, while combined doxorubicin with salinomycin treatment decreased ARK5 expression (Figure 4). Salinomycin treatment showed no obvious effects when used alone.

**Figure 5.**
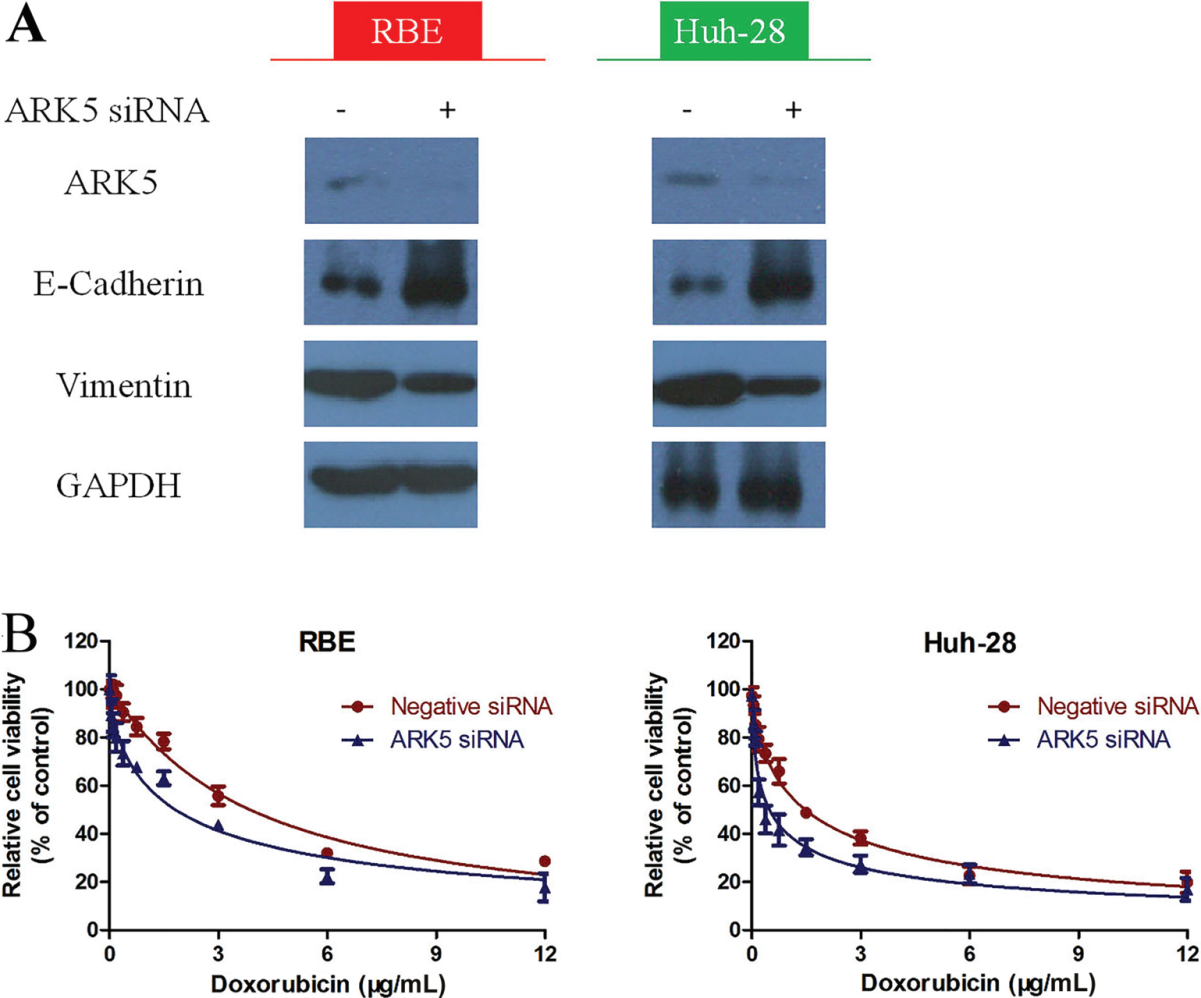
AMP-activated protein kinase family member 5 (ARK5) knockdown reverses epithelial-mesenchymal transition. *A*, Expression of E-cadherin and vimentin in RBE and Huh-28 cells following ARK5 suppression with siRNA. *B*, CCK-8 assay results of the viability of RBE and Huh-28 cells following doxorubicin (DOX) treatment after ARK5 siRNA interference.

We then investigated whether ARK5 is involved in the doxorubicin-induced EMT process. We used ARK5-siRNA to downregulate ARK5 expression in RBE and Huh-28 cells, and then monitored the expression of the EMT markers, E-cadherin and vimentin. In both cells lines, the epithelial marker E-cadherin was upregulated while expression of the mesenchymal marker vimentin decreased significantly (Figure 5A). Thus, salinomycin treatment may reverse doxorubicin-induced EMT by decreasing ARK5 expression.

Furthermore, in order to prove that ARK5 is involved in doxorubicin resistance, we downregulated the ARK5 expression with ARK5 siRNA in both cells, then treated with doxorubicin. The results showed that in RBE cells, the IC_50_ for doxorubicin was 1.820 μg/mL after ARK5 siRNA interference, which was significantly lower compared to negative siRNA interference (P<0.05). Similarly, the IC_50_ for doxorubicin in Huh-28 cells was 0.485 µg/mL with ARK5 siNRA interference, which was also significantly down regulated compared to negative siRNA interference (P<0.05; Figure 5B).

## Discussion

Cholangiocarcinoma remains difficult to detect in its early stages, which leaves patients with limited treatment options due to the late diagnosis and high rates of metastasis. In addition, response rates to chemotherapy in cholangiocarcinoma patients remain low, primarily due to the development of drug resistance (11). Chemoresistance (e.g., to doxorubicin) remains a big problem in the clinic, and may result from the induction of EMT in cancer cells. Indeed, the EMT process has previously been associated with invasion, metastasis, and chemoresistance in many malignancies, including cholangiocarcinoma (29–31). Likewise, our present study showed that treatment with doxorubicin induced the transformation of epithelial type cholangiocarcinoma cells into a mesenchymal type. Therefore, understanding how to reverse this EMT process at a mechanistic level to avoid chemoresistance is crucial for improving the outcome for cholangiocarcinoma patients.

Previous studies have reported that salinomycin could be useful in cancer chemotherapy (21–23), with one study indicating that it participates in the EMT process of cancer cells (23). Here, we demonstrated that treating cholangiocarcinoma cells with a combined therapy of doxorubicin and salinomycin enhanced the effect of the chemotherapy. The dosages of both drugs that were required to produce a cytotoxic effect decreased, indicating that these two drugs have a synergistic effect. In addition, salinomycin could reverse doxorubicin-induced EMT, as shown morphologically, as well as through the detection of EMT markers on cholangiocarcinoma cells. Moreover, we found that twist knockdown could block the synergistic effect of salinomycin and doxorubicin. Twist is considered an important transcription factor involved in EMT progress in cholangiocarcinoma (32), which is also essential in doxorubicin induced EMT (33). These results suggest salinomycin could enhance the effects of doxorubicin chemotherapy through reversing the EMT process.

As further evidence, we examined the effects of salinomycin and doxorubicin on ARK5 expression. ARK5 has previously been proven to be associated with invasion, metastasis and poor prognosis in breast cancer, colorectal carcinoma, non-small cell lung cancer, and cholangiocarcinoma (34–37). We found that after treatment with doxorubicin, cholangiocarcinoma cells had higher expression of ARK5, while salinomycin treatment could reserve this effect. Furthermore, ARK5 appears to regulate the expression of the EMT related markers E-cadherin and vimentin in cholangiocarcinoma cells; in particular, downregulation of ARK5 increased the expression of E-cadherin and decreases the expression of vimentin.

In conclusion, our study indicated that ARK5 expression may influence doxorubicin sensitivity through regulating the EMT process in cholangiocarcinoma cells. Furthermore, salinomycin could reverse the EMT process in cholangiocarcinoma cells by inhibiting ARK5 expression. Therefore, a combined treatment of salinomycin with doxorubicin could be used to enhance doxorubicin sensitivity in patients with cholangiocarcinoma.

## References

[1] Khan SA, Toledano MB, Taylor-Robinson SD (2008). Epidemiology, risk factors, and pathogenesis of cholangiocarcinoma. HPB.

[2] Siegel R, Naishadham D, Jemal A (2013). Cancer statistics, 2013. CA Cancer J Clin.

[3] Gatto M, Bragazzi MC, Semeraro R, Napoli C, Gentile R, Torrice A (2010). Cholangiocarcinoma: Update and future perspectives. Dig Liver Dis.

[4] Nathan H, Pawlik TM, Wolfgang CL, Choti MA, Cameron JL, Schulick RD (2007). Trends in survival after surgery for cholangiocarcinoma: a 30-year population-based SEER database analysis. J Gastrointest Surg.

[5] Zhang H, Yang T, Wu M, Shen F (2016). Intrahepatic cholangiocarcinoma: epidemiology, risk factors, diagnosis and surgical management. Cancer Lett.

[6] Yang L, Shan J, Shan L, Saxena A, Bester L, Morris DL (2015). Trans-arterial embolisation therapies for unresectable intrahepatic cholangiocarcinoma: a systematic review. J Gastrointest Oncol.

[7] Cillo U, Spolverato G, Vitale A, Ejaz A, Lonardi S, Cosgrove D (2015). Liver resection for advanced intrahepatic cholangiocarcinoma: a cost-utility analysis. World J Surg.

[8] Khan SA, Davidson BR, Goldin RD, Heaton N, Karani J, Pereira SP (2012). British Society of Gastroenterology. Guidelines for the diagnosis and treatment of cholangiocarcinoma: An update. Gut.

[9] Fabris L, Alvaro D (2012). The prognosis of perihilar cholangiocarcinoma after radical treatments. Hepatology.

[10] Zabron A, Edwards RJ, Khan SA (2013). The challenge of cholangiocarcinoma: Dissecting the molecular mechanisms of an insidious cancer. Dis Model Mech.

[11] Valle J, Wasan H, Palmer DH, Cunningham D, Anthoney A, Maraveyas A (2010). ABC-02 Trial Investigators. Cisplatin plus Gemcitabine versus Gemcitabine for Biliary Tract Cancer. N Engl J Med.

[12] Dibble CC, Cantley LC (2015). Regulation of mTORC1 by PI3K signaling. Trends Cell Biol.

[13] Leal P, Garcia P, Sandoval A, Buchegger K, Weber H, Tapia O (2013). AKT/mTOR substrate P70S6K is frequently phosphorylated in gallbladder cancer tissue and cell lines. Onco Targets Ther.

[14] Hoshino H, Miyoshi N, Nagai K, Tomimaru Y, Nagano H, Sekimoto M (2009). Epithelial-mesenchymal transition with expression of SNAI1-induced chemoresistance in colorectal cancer. Biochem Biophys Res Commun.

[15] Zhuo W, Wang Y, Zhuo X, Zhang Y, Ao X, Chen Z (2008). Knockdown of Snail, a novel zinc finger transcription factor, via RNA interference increases A549 cell sensitivity to cisplatin via JNK/mitochondrial pathway. Lung Cancer.

[16] Kurrey NK, Jalgaonkar SP, Joglekar AV, Ghanate AD, Chaskar PD, Doiphode RY (2009). Snail and slug mediate radioresistance and chemoresistance by antagonizing p53-mediated apoptosis and acquiring a stem-like phenotype in ovarian cancer cells. Stem Cells.

[17] Zong H, Yin B, Zhou H, Cai D, Ma B, Xiang Y (2014). Inhibition of mTOR pathway attenuates migration and invasion of gallbladder cancer via EMT inhibition. Mole Biol Rep.

[18] Cao Y, Liu X, Lu W, Chen Y, Wu X, Li M (2015). Fibronectin promotes cell proliferation and invasion through mTOR signaling pathway activation in gallbladder cancer. Cancer Lett.

[19] Tam KH, Yang ZF, Lau CK, Lam CT, Pang RW (2009). Inhibition of mtor enhance chemosensitivity in hepatocellular carcinoma. Cancer Lett.

[20] Meacham CE, Morrison SJ (2013). Tumor heterogeneity and cancer cell plasticity. Nature.

[21] Sachlos E, Risueno RM, Laronde S, Shapovalova Z, Lee JH, Russell J (2012). Identification of drugs including a dopamine receptor antagonist that selectively target cancer stem cells. Cell.

[22] Gupta PB, Onder TT, Jiang G, Tao K, Kuperwasser C, Weinberg RA (2009). Identification of selective inhibitors of cancer stem cells by high-throughput screening. Cell.

[23] Zhou Y, Liang C, Xue F, Chen W, Zhi X, Feng X (2015). Salinomycin decreases doxorubicin resistance in hepatocellular carcinoma cells by inhibiting the β-catenin/TCF complex association via FOXO3a activation. Oncotarget.

[24] Sanchez-Tillo E, Fanlo L, Siles L, Montes-Moreno S, Moros A, Chiva-Blanch G (2014). The EMT activator ZEB1 promotes tumor growth and determines differential response to chemotherapy in mantle cell lymphoma. Cell Death Differ.

[25] Terashita K, Chuma M, Hatanaka Y, Hatanaka K, Mitsuhashi T, Yokoo H (2016). ZEB1 expression is associated with prognosis of intrahepatic cholangiocarcinoma. J Clin Pathol.

[26] Kusunoki S, Kato K, Tabu K, Inagaki T, Okabe H, Kaneda H (2013). The inhibitory effect of salinomycin on the proliferation, migration and invasion of human endometrial cancer stem-like cells. Gynecol Oncol.

[27] Suzuki A, Kusakai G, Kishimoto A, Lu J, Ogura T, Lavin MF, Esumi H (2003). Identification of a novel protein kinase mediating Akt survival signaling to the ATM protein. J Biol Chem.

[28] Xu T, Zhang J, Chen W, Pan S, Zhi X, Wen L (2016). ARK5 promotes doxorubicin resistance in hepatocellular carcinoma via epithelial-mesenchymal transition. Cancer Lett.

[29] Sato Y, Harada K, Itatsu K, Ikeda H, Kakuda Y, Shimomura S (2010). Epithelial-mesenchymal transition induced by transforming growth factor-β1/Snail activation aggravates invasive growth of cholangiocarcinoma. Am J Pathol.

[30] Ryu HS, Chung JH, Lee K, Shin E, Jing J, Choe G (2012). Overexpression of epithelial-mesenchymal transition-related markers according to cell dedifferentiation: clinical implications as an independent predictor of poor prognosis in cholangiocarcinoma. Hum Pathol.

[31] Gu MJ, Choi JH (2014). Epithelial-mesenchymal transition phenotypes are associated with patient survival in intrahepatic cholangiocarcinoma. J Clin Pathol.

[32] Vaquero J, Guedj N, Clapéron A, Nguyen Ho-Bouldoires TH, Paradis V, Fouassier L (2017). Epithelial-mesenchymal transition in cholangiocarcinoma: From clinical evidence to regulatory networks. J Hepatol.

[33] Dai X, Ahn KS, Wang LZ, Kim C, Deivasigamni A, Arfuso F (2016). Ascochlorin enhances the sensitivity of doxorubicin leading to the reversal of epithelial-to-mesenchymal transition in hepatocellular carcinoma. Mol Cancer Ther.

[34] Chang XZ, Yu J, Liu HY, Dong RH, Cao XC (2012). ARK5 is associated with the invasive and metastatic potential of human breast cancer cells. J Cancer Res Clin Oncol.

[35] Shi L, Zhang B, Sun X, Lu S, Liu Z, Liu Y (2014). MiR-204 inhibits human NSCLC metastasis through suppression of NUAK1. Br J Cancer.

[36] Kusakai G, Suzuki A, Ogura T, Miyamoto S, Ochiai A, Kaminishi M (2004). ARK5 expression in colorectal cancer and its implications for tumor progression. Am J Pathol.

[37] Xiong X, Sun D, Chai H, Shan W, Yu Y, Pu L, Cheng F (2015). MiR-145 functions as a tumor suppressor targeting NUAK1 in human intrahepatic cholangiocarcinoma. Biochem Biophys Res Commun.

